# Improving curcumin bactericidal potential against multi-drug resistant bacteria via its loading in polydopamine coated zinc-based metal–organic frameworks

**DOI:** 10.1080/10717544.2022.2159587

**Published:** 2023-01-31

**Authors:** Abdul Jabbar, Khadija Rehman, Tooba Jabri, Tasmina Kanwal, Samina Perveen, Md Abdur Rashid, Mohsin Kazi, Saeed Ahmad Khan, Salim Saifullah, Muhammad Raza Shah

**Affiliations:** a H.E.J. Research Institute of Chemistry, International Center for Chemical and Biological Sciences, University of Karachi, Karachi, Pakistan; b School of Environmental and Biological Engineering, Nanjing University of Science and Technology, Nanjing, PR China; c Department of Pharmaceutics, College of Pharmacy, King Khalid University, Guraiger, Abha, Saudi Arabia; d Pharmacy Discipline, Faculty of Health, School of Clinical Sciences, Queensland University of Technology, Brisbane, Queensland, Australia; e Department of Pharmaceutics, College of Pharmacy, King Saud University, Riyadh, Saudi Arabia; f Department of Pharmacy, Kohat University of Science and Technology, Kohat, Pakistan; g Division of Molecular Pharmaceutics and Drug Delivery, College of Pharmacy, The University of Texas at Austin, Austin, Texas, USA; h Pakistan Forest Institute, Peshawar, Pakistan

**Keywords:** metal-organic frameworks, Polydopamine coating, characterization, curcumin loading, bactericidal potential

## Abstract

Multi-drug resistant (MDR) bactearial strains have posed serious health issues, thus leading to a significant increase in mortality, morbidity, and the expensive treatment of infections. Metal-organic frameworks (MOFs), comprising metal ions and a variety of organic ligands, have been employed as an effective drug deliveryy vehicle due to their low toxicity, biodegradability, higher structural integrity and diverse surface functionalities. Polydopamine (PDA) is a versatile biocompatible polymer with several interesting properties, including the ability to adhere to biological surfaces. As a result, modifying drug delivery vehicles with PDA has the potential to improve their antimicrobial properties. This work describes the preparation of PDA-coated Zn-MOFs for improving curcumin’s antibacterial properties against S. aureus and E. coli. Powder X-ray diffraction (P-XRD), FT-IR, scanning electron microscopy (SEM), and DLS were utilized to characterize PDA-coated Zn-MOFs. The curcumin loading and in vitro release of the prepared MOFs were also examined. Finally, the MOFs were tested for bactericidal ability against E. coli and S. aureus using an anti-bacterial assay and surface morphological analysis. Smaller size MOFs were capable of loading and releasing curcumin. The findings showed that as curcumin was encapsulated into PDA-coated MOFs, its bactericidal potential was significantly enhanced, and the findings were further supported by SEM which indicated the complete morphological distortion of the bacteria after treatment with PDA-Cur-Zn-MOFs. These studies clearly indicate that the PDA-Cur-Zn-MOFs developed in this study are extremely promising for long-term release of drugs to treat a wide range of microbial infections.

## 1. Introduction

Metal-organic frameworks (MOFs) are porous materials having crystalline morphology comprised of organic molecules coordinated with metal ions that can be used to produce three-dimensional structures with unique properties. These materials generally contain active components, the potential for pore functionalization, a high surface-to-volume ratio, and a large pore volume (Furukawa et al., 2013; Shah et al., 2022). MOFs have displayed remarkable properties in numerous scientific domains, including gas storage and separation, electrochemistry, sensing, and catalysis, and their potential applications in the field of biomedicine are gaining considerable attention (Li et al., 2009; Horcajada et al., 2012; Morozan and Jaouen, 2012; Baumann et al., 2019; Meteku et al., 2021). The compositional diversity of these materials permits the development of theranostics, contrast agents, and drug delivery systems with an acceptable toxicity profile (Horcajada et al., 2006; 2010; Rojas et al., 2019). Zinc (II) is among the most commonly employed cations in MOFs design. Secondary building units, typically endless rod-like structures or discrete clusters, have evolved as a result of the wide range of coordination geometries enabled by its electrical arrangement, which ranges from four- to six-sided polyhedral (Tranchemontagne et al., 2009; Schoedel et al., 2016). Zinc (II) is a component of several important enzymes and transcription factors, making it an essential nutrient with a daily intake of 10 mg (Shankar and Prasad, 1998; Trumbo et al., 2001). It plays an important role in oxidative stress response, immunological response, aging, apoptosis, DNA replication and damage repair, and protein synthesis, including collagen production (Chasapis et al., 2020). Zinc’s toxicity has also been investigated extensively, and the oral lethal dose for humans is approximately 3 g/kg of body weight (Roney et al., 2006; Plum et al., 2010). Zn is also an antioxidant mineral because, as a cation, it is redox inactive and does not promote the formation of reactive oxygen species (Shankar and Prasad, 1998; Chasapis et al., 2020). Antibacterial efficacy is affected by concentration and contact time. Zinc’s antimicrobial effects are due to direct contact with microbial membranes, coordination with nucleic acids, and respiratory system inhibition (Pasquet et al., 2014).

Polydopamine (PDA) has drawn significant interest in a variety of sectors due to its adaptable hydrophilicity, biocompatibility, and adhesion capability and has been utilized to modify the surface of porous materials without altering their pore shape (Hebbar et al., 2016; Hou et al., 2021; Jadidi et al., 2022). Self-polymerization of dopamine in a mildly alkaline tris-buffer solution modifies different substrates by adding water-holding phenolic OH and NH moieties (Barclay et al., 2017). In addition to its antifungal and antibacterial properties against a wide range of microorganisms, PDA has also been used to treat cancer (Zhou et al., 2021). The development of a simple shaking-assisted process has facilitated the fabrication of roughened PDA (rPDA) coatings on a variety of substrates (Singh et al., 2021). When compared to the control coatings, rPDA coatings exhibit much higher antimicrobial potential against a variety of bacteria, both Gram-negative and Gram-positive, without the use of an external antibacterial agent (Su et al., 2016). PDA coatings can be utilized to manipulate antibacterial activity by utilizing a range of buffers, as the buffer selection can influence the amount of a certain functional group present in the PDA coating (Patel et al., 2018). PDA coatings mediated by sodium hydroxide and tris were found to have higher antibacterial activity than coatings mediated by phosphate-buffered saline (PBS) and sodium bicarbonate (Patel et al., 2018). This difference could be explained by the presence of an increased number of surface hydroxyl groups in the latter. It was discovered that different chemistries had different effects on the physicochemical characteristics and morphology of the PDA coatings, which in turn had different effects on the antibacterial or antifouling activities of the coatings (Zhou et al., 2014). Recently, PDA coating on the surface of nanocarriers has received considerable interest in the development of nanocarriers, particularly in the preparation of MOFs. This is due to the availability of several functional groups in PDA, namely amine and catechol, which make it a suitable platform for the co-ordination of metal ions (Yang et al., 2016). In a similar manner, Jiajing Zhou et al., designed PDA coated nanohybrids that consisted of a MOFs shell and a nanoparticle core (Yang et al., 2016). In a study published by Isabelle I. Niyonshuti et al., the PDA-AgNPs were developed with a controllable PDA coating thickness to examine the impact of the surface on the antibacterial activity of the AgNPs (Niyonshuti et al., 2020). Therefore, PDA has been selected as the surface coating material because of its excellent adhesive properties.

Infectious diseases caused by bacteria are one of the world’s leading health concerns, affecting millions of individuals each year. Antibiotic-resistant bacterial infections are becoming more common and pose a severe threat to public health (Koch et al., 2014; Willyard, 2017; Cheng et al., 2019). Antibiotics are the most extensively used and efficient way of preventing and treating bacterial diseases (Spellberg et al., 2013). However, antibiotic overuse and misuse have resulted in the emergence and spread of antibiotic-resistant microorganisms (Brown and Wright, 2016). Moreover, bacterial colonization and biofilm formation by sessile microbial populations represent a significant clinical problem (Iwase et al., 2010). Therefore, alternative therapeutic approaches for bacterial infections and biofilms are urgently required to prevent the evolution of antibiotic resistance. Antibacterial activity of curcumin (CUR) has been discovered at nontoxic and safe levels in recent years (Zorofchian Moghadamtousi et al., 2014; Shome et al., 2016). There are numerous hypotheses regarding the mechanism by which CUR destroys bacteria. According to Tyagi et al., CUR can inhibit bacterial growth by rupturing the bacterial membrane (Tyagi et al., 2015). Lee et al., studied the CUR’s antibacterial mechanism, which they found to be mediated by an apoptotic-like response (Yun and Lee, 2016). However, due to CUR’s poor water solubility, fast breakdown, and poor oral bioavailability, its therapeutic potential is limited (Mahmood et al., 2015; Yallapu et al., 2015). The development of an efficient nano-carrier technology for the purpose of resolving issues associated with CUR is extremely desirable in order to enhance its antibacterial activity.

In this study, PDA-coated CUR-loaded MOFs (PDA-CUR-Zn-MOFs) were prepared in order to improve the efficacy of CUR and increase its action against bacteria. PDA was employed to modify the surface of MOFs in order to enhance their adherence to bacterial cells, which in turn inhibits the growth of bacteria. The PDA-CUR-Zn-MOFs were analyzed using various techniques, including FT-IR, SEM, Powder X-ray diffraction, and DLS. The antibacterial activity of the PDA-CUR-Zn-MOFs was tested on two different bacterial strains. In addition, SEM was performed to investigate the attachment of PDA-CUR-Zn-MOFs to bacteria and to determine the structural and morphological variations in the bacteria after treatment with PDA-CUR-Zn-MOFs.

## 2. Experimental

### 2.1. Materials and methods

For this experiment, analytical-grade solvents were bought from Sigma Aldrich (Germany). Rueddel-deHaen (Germany) provided the 99.9% pure Zn(OAc)2.2H2^.^Moreover, terephthalic acid, dopamine, and 3-(4, 5-dimethylthiazol-2-yl)-2, 5-diphenylthiazol-2-yl)-2, 5-diphenyltetrazolium bromide (MTT) were acquired from Sigma Aldrich (Germany). The tryptic soya agar (TSA) and Mueller Hinton broth (MHB) were provided by Oxoid, UK. Bosch Pharmaceuticals (Pvt) Ltd. in Pakistan graciously supplied the CUR that was utilized in this investigation.

### 2.2. Preparation of zinc-terephthalate MOFs (Zn-MOFs)

**Figure 1.**
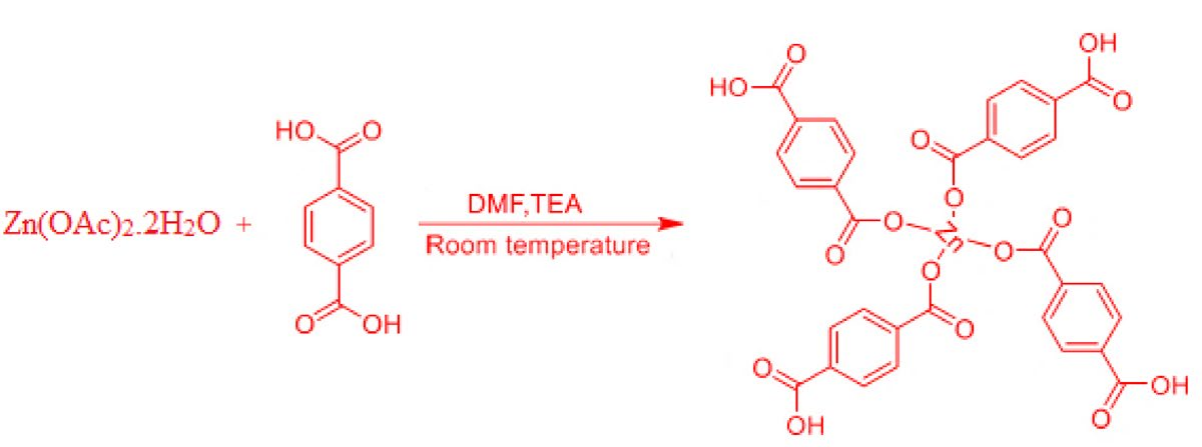
Schematic illustration of the synthesis of Zn-MOFs.

The Zn-MOFs were synthesized using a previously published method (Ghasemzadeh et al., 2018), with a few modifications. Briefly, one mmol of terephthalic acid and two mmol of zinc acetate dihydrate were dissolved in ten mL of dimethylformamide (DMF), which was then stirred and mixed. After 2.5 hours of stirring at room temperature, 0.3 mL of triethylamine was mixed to the solution. The orange-yellow precipitates were obtained by centrifugation for 10 minutes at 5000 rpm, washed with ethanol and DMF to separate any residual solvent, and then dried in a vacuum oven. Figure 1 depicts a schematic representation of Zn-MOFs synthesis (Tella et al., 2020).

### 2.3. Encapsulation of curcumin in Zn-MOFs (CUR-Zn-MOFs)

In order to encapsulate CUR, 60 mg of Zn-MOFs dispersion in distilled water (6 mL) and 30 mg of CUR solution in methanol (4 mL) were mixed. The mixture was then stirred at 200 rpm for 24 hours. Thus, the CUR was encapsulated in Zn-MOFs. The prepared CUR-Zn-MOFs were then collected by centrifugation at 10,000 rpm for 15 minutes. The free CUR was measured at 420 nm in the supernatant utilizing a UV-Vis spectrophotometer (UV-240, Shimadzu, Japan). The % drug encapsulation efficiency (%DEE) was measured utilizing the formula below (Kanwal et al., 2019).

$$\%\mathrm{DEE}=\frac{\text{Total amount of drug}-\text{Amount of free drug in the supernatant}}{\text{Total amount of drug}}\;\times 100$$

### 2.4. Preparation of PDA coated CUR-Zn-MOFs (PDA-CUR-Zn-MOFs)

**Scheme 1.**
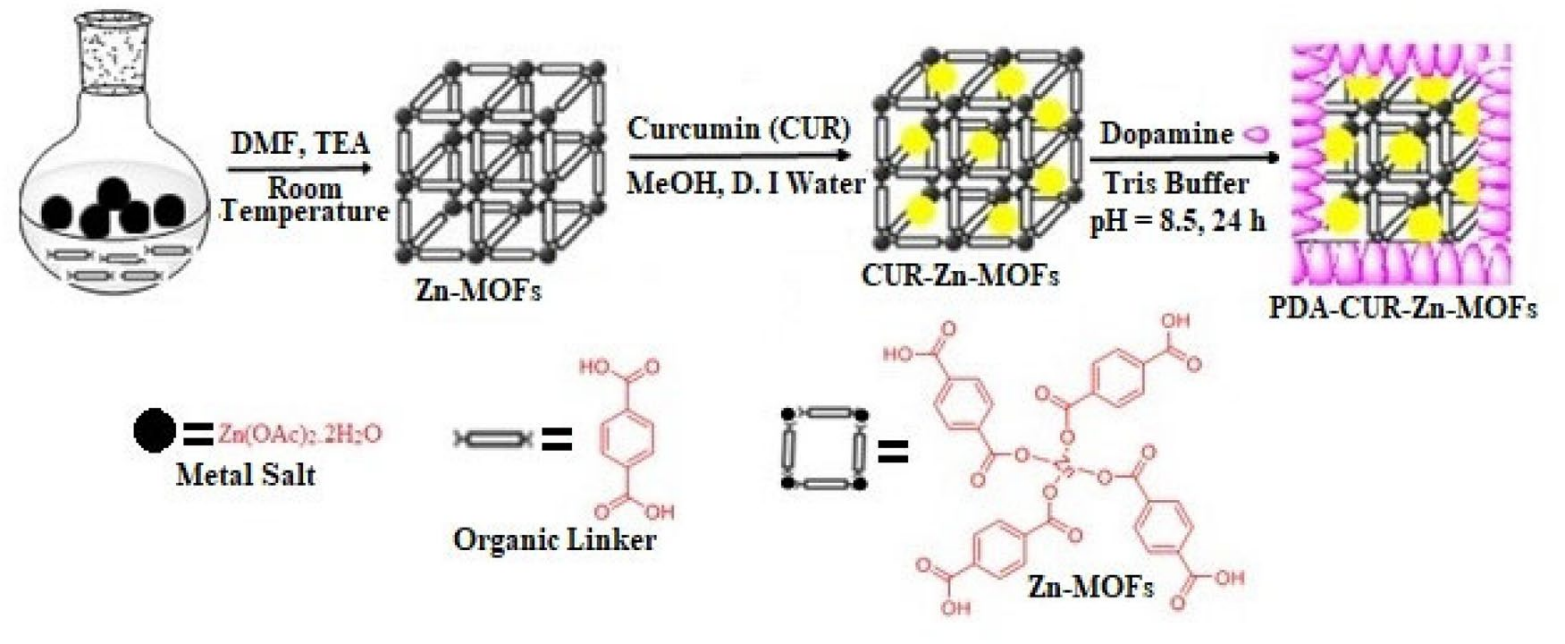
Synthesis routs for the preparation of PDA-CUR-Zn-MOFs.

A previously published method was adopted to generate PDA-coated CUR-Zn-MOFs, with minor changes (Shan et al., 2020). Briefly, 19 mg of dopamine was dissolved in a Tris buffer solution (10 mM, pH 8.5) while stirring. The solution was stirred for 24 hours after adding 25 mg of Cur-Zn-MOFs. PDA-CUR-Zn-MOFs were produced by centrifuging the resulting solid for 10 minutes at 10,000 rpm, thoroughly washing it using water, and then oven-dried it for 6 hours at 50 °C. The schematic representation for the preparation of PDA-CUR-Zn-MOFs is depicted in Scheme 1.

### 2.5. Characterization

#### 2.5.1. Zeta potential, size, polydispersity index (PDI), and surface morphology

After being diluted with distilled water, the samples were examined in triplicate on a zeta-sizer (Nano ZS90, Malvern Instruments, UK) to evaluate particle size, zeta potential, and PDI at 25 °C and a scattering angle of 90. SEM was performed to evaluate the surface morphology of the developed MOFs. During the SEM analysis, samples were gold-coated at temperatures of up to 300°A, and then images were captured using a Japanese JEOL microscope (Model number JSM-6380A).

#### 2.5.2. FTIR analysis

FT-IR analysis of Zn-MOFs, dopamine, PDA-CUR-Zn-MOFs, CUR, and CUR-Zn-MOFs was performed by an infrared spectrophotometer (Shimadzu, Kyoto). The samples were mixed with KBr pellets and compressed to a pressure of 1.38 × 10^3^ kPa to form disks. Between 400 and 4000 cm^−1^, the IR spectra of the formed disks were recorded.

#### 2.5.3. Powder X-ray diffraction (P-XRD)

The purity, size, and crystallinity of the prepared Zn-MOFs were examined using the P-XRD method. From 5° to 60° (2θ), diffraction patterns were evaluated utilizing an X-ray diffraction instrument (Axios Petro, PANalytical, Co Kα, λ = 1.79021) with Cu-Kα irradiation.

#### 2.5.4. Analysis of in vitro drug release

In order to perform the drug release study, PDA-CUR-Zn-MOFs (5 mg) were dispersed in sterile water and then poured into a dialysis bag. The dialysis bag was then immersed in 30 mL of buffer solution with a pH of 6.8 in an Erlenmeyer flask. At predetermined intervals, 2 mL of the dissolution medium were separated and replaced with 2 mL of a fresh solution. A UV-Vis-Spectrophotometer was employed to measure the released CUR in the obtained samples at 420 nm (Motakef-Kazemi et al., 2014).

### 2.6. Antibacterial assay

#### 2.6.1. Bacterial strains

Two ATCC bacterial strains, S. aureus (ATCC 6538) and E. coli (ATCC 10536) were derived from the organism under study, and their antibacterial activity was evaluated in the laboratory. The stock cultures were stored in TSA at 4° C. Before the antibacterial analysis, the bacterial strains were subcultured for 24 hours on fresh, appropriate agar. Several single-microbe colonies were injected into an inert MHB to create inocula. To confirm that the bacterial cells in suspension had reached a final density of 5 × 10^5^ cfu/mL, viable cell counts were performed. In 1 mL of solution, there were 10^5^ colony-forming units (cfu), which is the infectious dose for the majority of bacteria.

#### 2.6.2. Assessment of minimum inhibitory concentration (MIC)

As described previously (Khan et al., 2021), a Tetrazolium microplate assay was carried out to measure the MIC of both the reference materials and the bacteria that were subjected to the standard test. The experiment was conducted on a transparent 96-well microliter plate. Bacterial cell suspensions (5x10^5^cfu/mL) of S. aureus and E. coli were used to inoculate the wells immediately after harvest. Zn-MOFs, CUR, PDA-CUR-Zn-MOFs, and CUR-Zn-MOFs were serially diluted in MBH from 250 µg to 10 µg, and 200 µl of each concentration were then added in triplicate wells before the mixture was incubated at 37 °C ± 0.5 for 18 to 24 hours. Following that, MTT (50 µl, 0.2 mg/mL) was put to all wells and incubated at 37 °C for 30 min. A bacterial culture served as the positive control in this experiment, while DMSO served as the negative control. After DMSO was added, the concentration of the dye was determined utilizing a spectrophotometer with a reference wavelength of 650 nm and absorbance at 570 nm (indicating the inhibition of bacterial growth) (Sarkar et al., 2007).

$$\mathrm{IC}50=\frac{O.D.\text{ in Control}-\;O.D.\text{ of test }}{O.D.\text{ in control}}\;\times 100$$

#### 2.6.3. Minimum biofilm inhibitory concentration (MBIC) determination

Microplate experiments were performed to compare the antibiofilm abilities of PDA-CUR-Zn-MOFs, CUR-Zn-MOFs, Zn-MOFs, and CUR against E. coli and S. aureus. The aforementioned prepared substances were diluted in the same manner as previously stated and then placed in a 96-well flat bottom plate (Corning, USA). An inoculum of 5x10^5^ CFU mL^−1^ of bacteria was injected into all wells except the well containing the control broth. Plates were stained after an overnight incubation at 37 °C to identify biofilm formation (O’Toole et al., 1999). To remove any remaining planktonic cells, the plates were cleaned three times with sterile water and stained for 20 minutes with crystal violet at a concentration of 0.1% (w/v). To eliminate the crystal violet that had been retained by the biofilms, the plates were rinsed again and then treated with a 30% (v/v) solution of glacial acetic acid. The microplate reader (Tecan, USA) determined the plates’ absorbance at 590 nm. The following formula was employed to determine the percentage of inhibition of biofilms.

$$\text{\% biofilm inhibition }=\frac{O.D.\text{ in Control}-\;O.D.\text{ of test }}{O.D.\text{ in control}}\;\times 100$$

#### 2.6.4. Analysis of the surface morphology

The surface morphology of biofilms was studied using SEM. Changes in the morphology of exponentially growing E. coli and S. aureus cell suspensions (5x10^5^ CFU/mL) in response to a test samples were observed. MBIC microliter plates with the appropriate wells were used to collect the samples (5–10 µL), and drug-free cells worked as the control. As previously mentioned, the cells were pelleted and then fixed in a glutaraldehyde (2%) solution at 4 °C for 2 hours. The cells were dehydrated utilizing increasing concentrations of alcohols. After applying a gold coating, samples were analyzed utilizing a JSM-6380A SEM.

### 2.7. Statistical analysis

All experiments were performed in three times, and the results were given as the mean ± SEM.

## 3. Result and discussion

### 3.1. Characterization

#### 3.1.1. Size, zeta-potential and PDI

**Figure 2.**
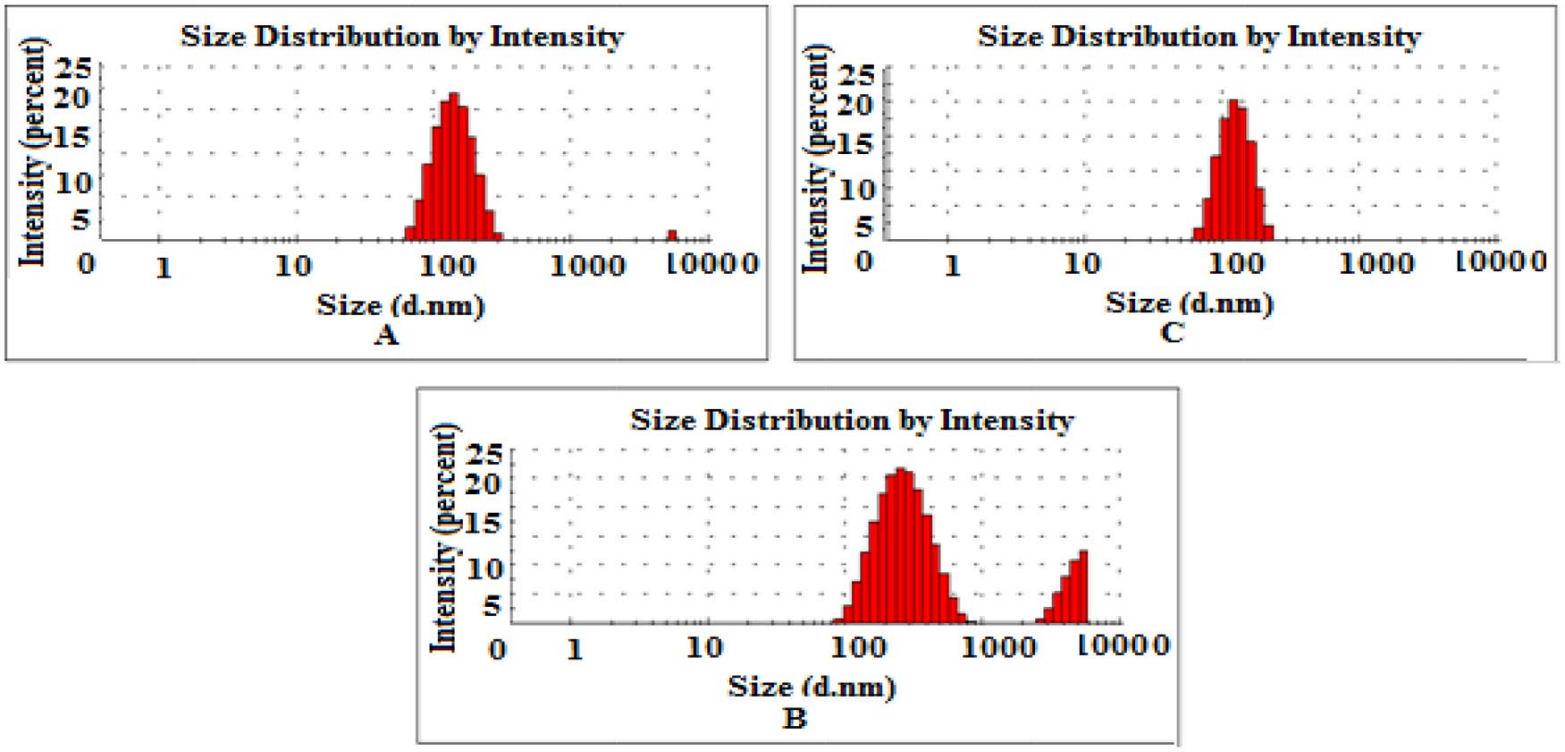
Size distribution of Zn-MOFs (A), CUR-Zn-MOFs (B), and PDA-CUR-Zn-MOFs (C).

**Figure 3.**
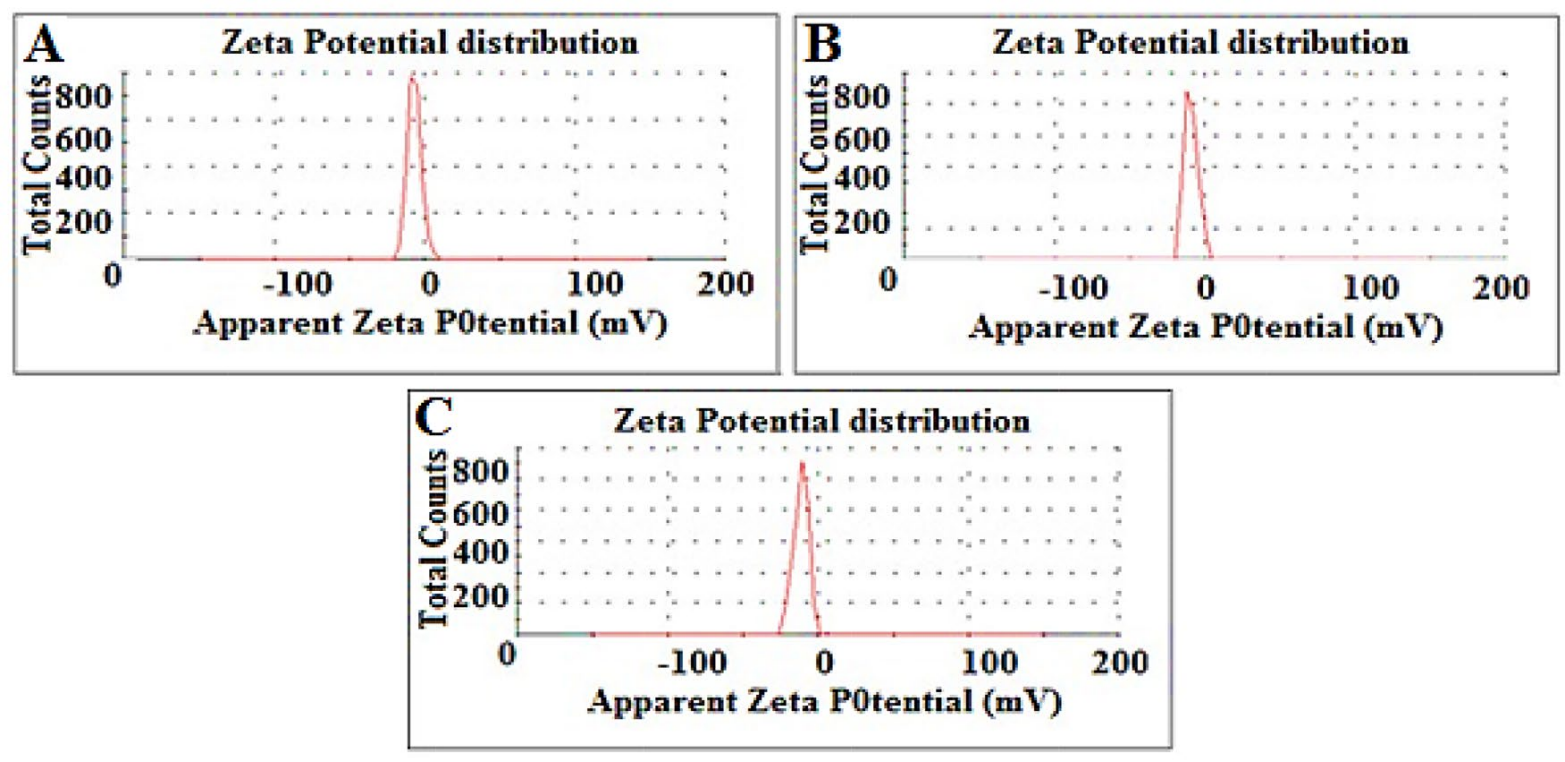
Zeta potential of Zn-MOFs (A), CUR-Zn-MOFs (B), and PDA-CUR-Zn-MOFs (C).

DLS was performed to determine the PDI, size and zeta potential of PDA-CUR-Zn-MOFs, CUR-Zn-MOFs, and Zn-MOFs. Particle size has a direct effect on the physical stability and therapeutic efficacy of encapsulated drugs, making it one of the most crucial and essential component of drug-delivery pharmacological formulations. MOFs are a subset of the larger category of porous crystalline nanomaterials, and the size of these materials can be precisely controlled by the fabrication method and solvent. It has been demonstrated that DMF creates particles that are even smaller than methanol and water (Khan et al., 2021). Cross-linkers utilized during production make MOFs more amenable to dissolution in DMF (Chalati et al., 2011). The particle size of Zn-MOFs was observed to be 200.9 ± 18 nm with a PDI of 0.233 ± 0.08; after CUR loading and PDA coating, the particle size increased to 220.8 ± 14 nm with a PDI of 0.317 ± 0.02 and 294.7 ± 04 nm with a PDI of 0.329 ± 0.064, as shown in Table 1 and Figure 2. When the CUR was encapsulated and PDA was coated on the Zn-MOFs, the PDI increased, indicating the MOFs’ uneven CUR loading and PDA coating. The Zeta potential is also crucial to drug delivery vehicles because it reveals information about the particles’ total net charge. The zeta potential of Zn-MOFs was determined to be −7.50 ± 0.04 due to the use of terephthalic acid as a cross-linker during their production. The surface charges of Zn-MOFs were found to increase slightly after PDA coating and CUR encapsulation (-9.38 mV ± 0.06 and −11.4 mV ± 0.08, respectively). Increases in the zeta potential value are anticipated to improve the physical stability of MOFs because particles with identical increased charges are believed to repel one another and remain suspended (Figure 3). In addition, coating by PDA ensures that the drug remains intact within Zn-MOFs.

**Table 1.** Size, PDI, Zeta potential, and encapsulation efficiency of prepared Zn-MOFs.

Test Sample	Size (nm)	PDI	Zeta Potential (mV)	% EE
Zn-MOFs	200.9 ± 18	0.233 ± 0.08	−7.50 ± 0.04	–
CUR-Zn-MOFs	220.8 ± 14	0.317 ± 0.02	−9.38 ± 0.06	80.04 ± 1.46%
PDA-CUR-Zn-MOFs	294.7 ± 04	0.329 ± 0.064	−11.4 ± 0.08	75.66 ± 2.24%

#### 3.1.2. Surface morphology

**Figure 4.**
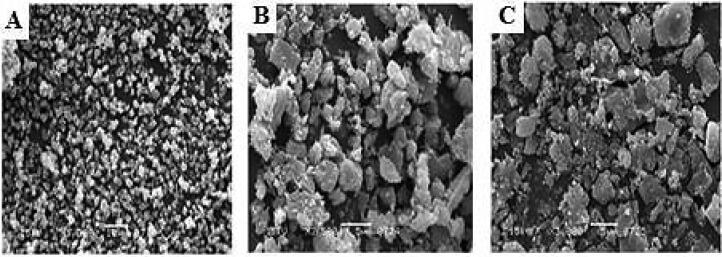
SEM images of Zn-MOFs (A), CUR-Zn-MOFs (B), and PDA-CUR-Zn-MOFs (C).

The SEM was utilized to examine the surface morphology of the prepared Zn-MOFs. Before drug loading, the Zn-MOFs displayed a smooth, crystalline surface (Figure 4A). Figure 4B demonstrates that the surface roughened slightly after drug loading and became entirely rough after PDA coating (Figure 4C). The drug experiences considerable morphological changes during loading and PDA coating, which may be suggestive of a partial breakdown of the framework. Drug-loaded MOFs had a more inflated morphology than empty MOFs, suggesting that the drug had been entrapped into the holes of the MOFs. These findings also demonstrated that drug-loaded MOFs were less porous and more densely filled than empty MOFs.

### 3.2. Drug encapsulation efficiency

The encapsulation of drugs in the developed drug-delivery carrier guarantees the delivery of therapeutics to the selective point with increased efficacy. Since the entrapment occurs primarily in the tunnels and cages of MOFs, the incorporation of the drug candidate in those structures is essentially determined by their surface area (Shen et al., 2017). The CUR loaded content into the prepared Zn-MOFs was 80.04 ± 1.46%. The encapsulation mechanism in the Zn-MOFs may require the adsorption of CUR on their surfaces or the inclusion in their holes through secondary interactions (hydrogen bonding and pi–pi stacking). After being coated with PDA, Zn-MOFs’ encapsulation efficiency slightly decreased (Table 1) which may be associated with the elimination of CUR that were weakly bound with Zn-MOFs during the PDA functionalization process.

### 3.3. FT-IR analysis

**Figure 5.**
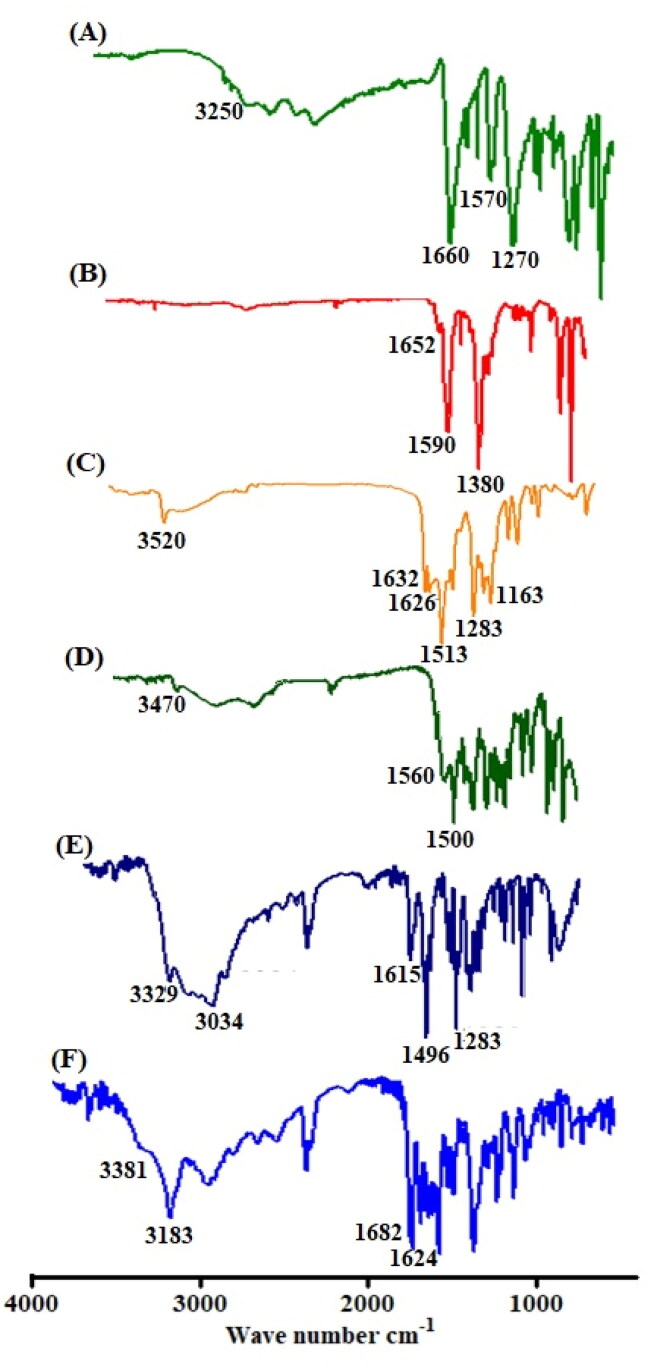
FTIR spectrum of terepthalic acid (A), Zn-MOFs (B), curcumin (C), CUR-Zn-MOFs (D), Dopamine (E), and PDA-CUR-Zn-MOFs (F).

The CUR loading, PDA coating, and Zn-MOFs synthesis were evaluated by FT-IR analysis. In the FT-IR spectrum of terepthalic acid, an absorption peak at 3250 cm^−1^ region corresponds to the O-H group and a significant absorption peak was observed at 1660 cm^−1^ corresponds to the C = O group of carboxylic acid (Figure 5A). The FT-IR spectrum of prepared Zn-MOFs did not exhibit the significant peak of terephthalic acid at 3250 cm^−1^ because of coordination with zinc. Moreover, after coordination with Zn, a little shift in absorption frequency from 1660 cm^−1^ to 1652 cm^−1^ was seen. The FT-IR spectra of CUR exhibited the typical peaks of O-H and C = O at 3520 cm^−1^ and 1626 cm^−1^, respectively, clearly demonstrating the interaction of CUR with the produced Zn-MOFs (Figure 5C). After being incorporated in Zn-MOFs, the significant peak of Zn-MOFs was slightly moved to 3450 cm^−1^ along with decreased intensity. In addition, the presence of CUR in the Zn-MOFs was confirmed by the appearance of the significant peak of C = O of CUR in the loaded Zn-MOFs (Figure 5D). The O-H signal was occurred at 3329 cm^−1^ in the FTIR spectrum of dopamine, while benzene ring was identified at 1615 cm^−1^ (Figure 5E). As shown in Figure 5F, the corresponding functional groups were submerged in the surface coating process, as the normal dopamine peak at 3329 cm^−1^ was disappeared whereas a prominent peak at 3183 cm^−1^ was found after modification.

### 3.4. Powder-XRD

**Figure 6.**
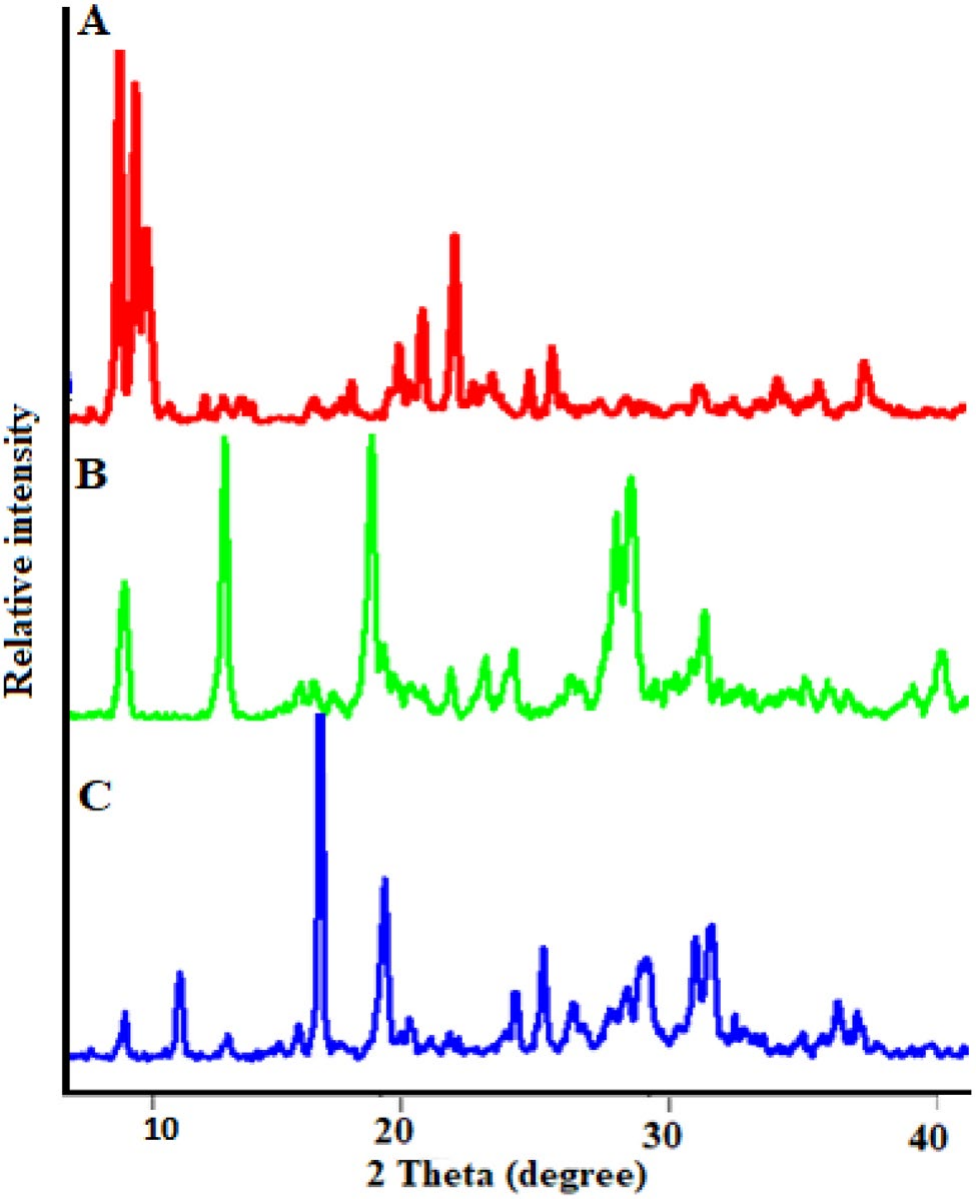
P-XRD of the synthesized Zn-MOFs (A), CUR-Zn-MOFs (B), and PDA-CUR-Zn-MOFs (C).

Powder-XRD is considered as an efficient technique to examine the crystallinity of materials. The P-XRD spectrum of Zn-MOFs exhibited the high intensity Bragg diffraction peaks at 2θ = 7.67°, 18.17°, 21.26°, 26.16°, 32.76° and 37.14° (Figure 5A) as reported previously (Dikio and Farah, 2013). Upon CUR loading, some structural changes appeared in the XRD spectrum with the prominent peak at 2θ = 18.98° and 27.81° attributing to the presence of crystalline CUR that are adsorbed on the surface of MOFs structure as reported previously (Chen et al., 2018). Furthermore, some changes in the intensities ratio of Bragg reflections can be observed, most likely because of changes in interatomic distances and bond angles, or the preferential orientation of crystallites imposed during the drug loading process and filling of pores by CUR, resulting in a partial loss of crystallinity, as shown in Figure 5B (Motakef-Kazemi et al., 2014; Blanita et al., 2015). Interestingly, the structure of CUR-Zn-MOFs was almost unaffected by the PDA coating, but the intensity of the characteristic peak was reduced that are showing the change in crystallinity (Figure 5C) which is primarily associated with the formation of PDA layer on the surface of MOFs structure (Liu et al., 2018). Hence, the observed results revealed the semicrystalline structure for PDA-CUR-Zn-MOFs and CUR-Zn-MOFs (Figure 6).

### 3.5. Analysis of in vitro drug release

**Figure 7.**
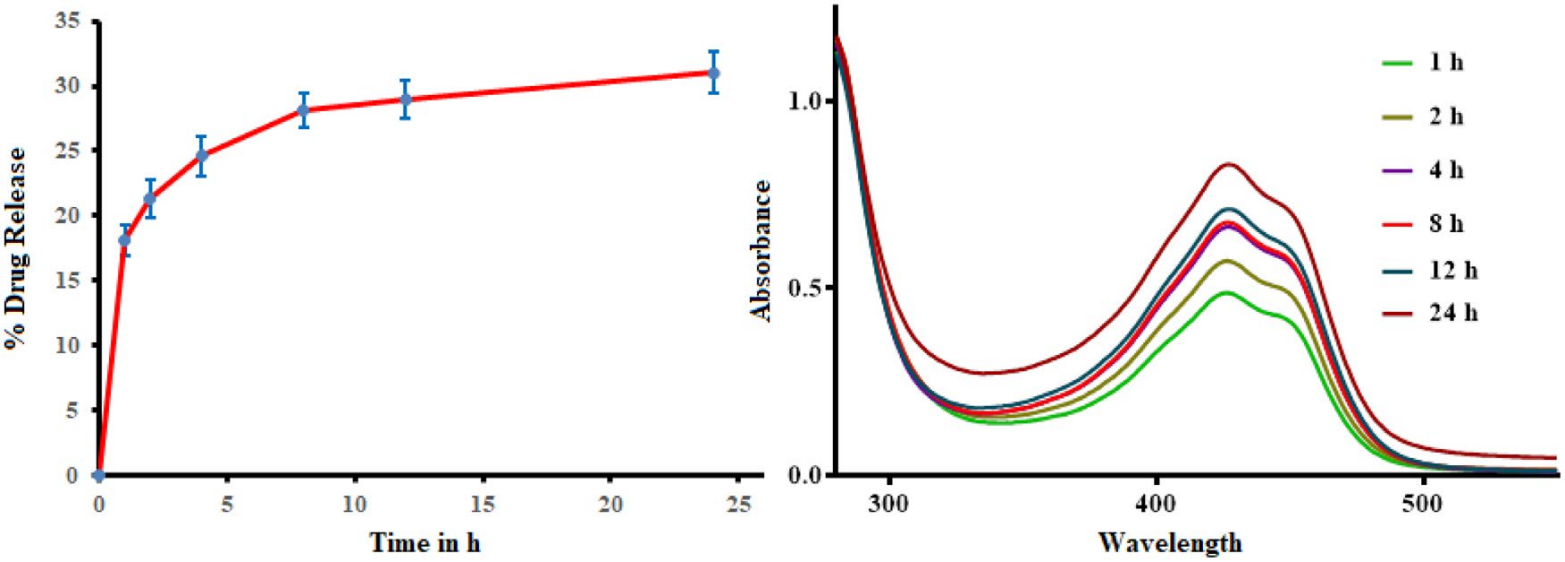
In vitro release profile of PDA-CUR-Zn-MOFs at pH 6.8.

Figure 7 shows the drug release pattern conducted at physiological pH of 6.8. The prepared PDA-CUR-Zn-MOFs exhibited an initial release of 17.45 ± 5.64% CUR after 1 h, followed by controlled release behavior for 24 h with a maximum release of 33.12 ± 6.10%. Initial rapid release may be affiliated to weakly-bound CUR diffusing across the surface of Zn-MOFs. While the CUR incorporated in the pores and linked through interaction with the aromatic ring promoted the extended-release behavior. The release profile of PDA-CUR-Zn-MOFs suggested that it may be suitable for applications that required a high initial dose followed by a maintained release.

### 3.6. Antibacterial analysis

Tetrazolium microplate testing was utilized for each sample to assess the antibacterial activity in terms of MIC, Half-maximal Inhibitory Concentration (IC_50_), and MBIC values. MIC is defined as the lowest concentration that effectively stops the observable growth of bacteria (Rehman et al., 2021). The concentration at which 50% of the bacteria are inhibited is referred to as the IC_50_, while the MBIC is the minimum concentration of the antibacterial agent that prevents the initial production of biofilm (Mohamed et al., 2018; Ghaffar et al., 2019). The results of the provided terms have been thoroughly discussed below.

#### 3.6.1. Determination of MIC value

**Figure 8.**
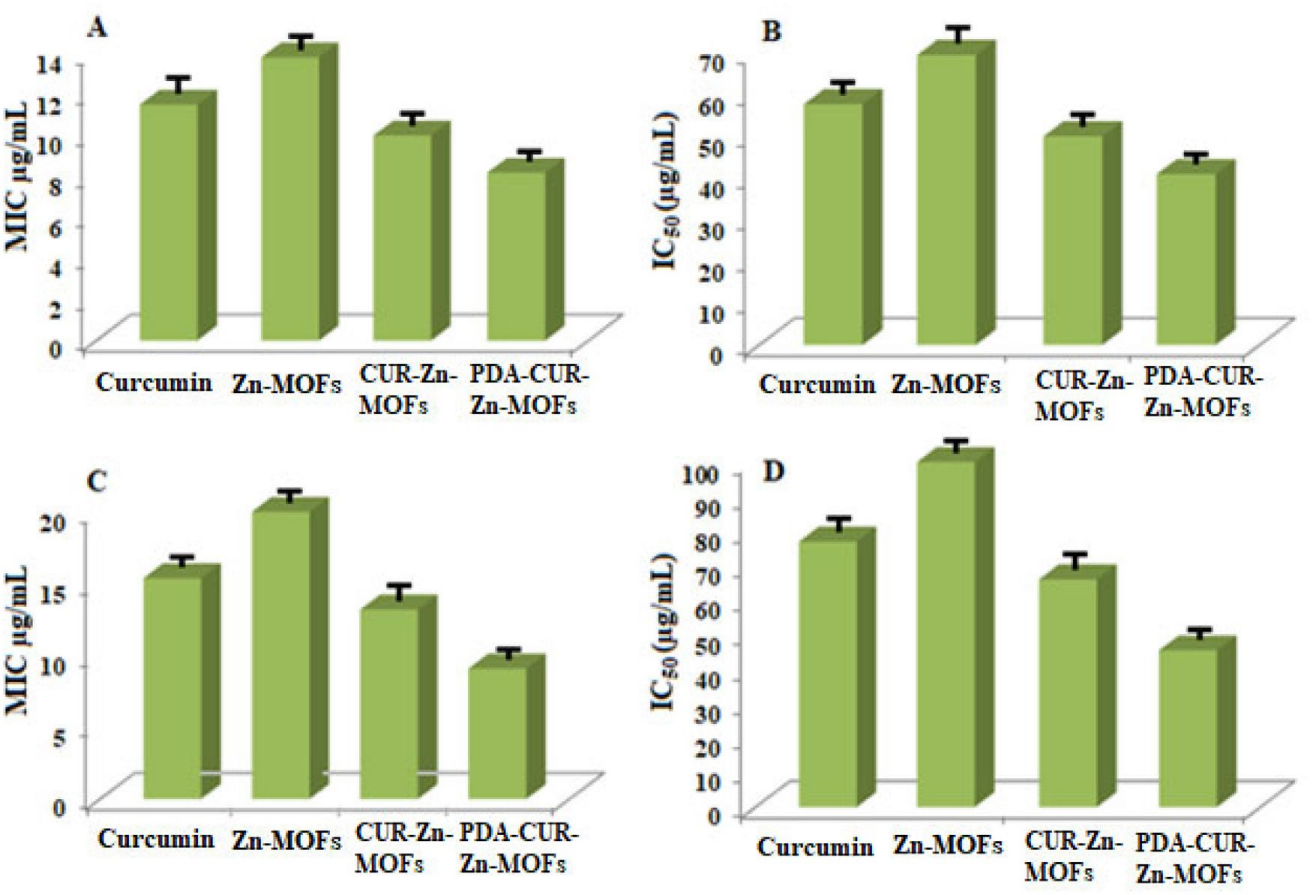
MIC and IC50 value of CUR, Zn-MOFs, CUR-Zn-MOFs and PDA-CUR-Zn-MOFs against S. aureus (A, B), and against E. coli (C, D).

A tetrazolium microplate assay was employed to evaluate the MIC for each sample. CUR had a MIC of 11.5 ± 0.5 µg/mL and Zn-MOFs had a MIC of 13.8 ± 0.8 µg/mL against S. aureus, but when the bacteria was treated with CUR-loaded Zn-MOFs, the MIC was reduced to 10 ± 0.4 µg/mL. Additionally, the MIC value for S. aureus was further reduced to 8.18 ± 0.6% µg/mL after treatment with PDA-CUR-Zn-MOFs, (Figure 8A). CUR’s IC_50_ against S. aureus cells was determined to be 57.69 ± 0.5 µg/mL, while Zn-MOFs exhibited an IC_50_ of 69.44 ± 0.8 µg/mL. As depicted in Figure 8B, the value of CUR-Zn-MOFs against S. aureus was reduced to 50 ± 0.4 µg/mL, and it was further decreased to 40.9 ± 0.6 µg/mL after modification with PDA.

MIC values for CUR and Zn-MOFs against E. coli were 15.38 ± 0.5% and 20 ± 0.5 µg/mL respectively. As displayed in Figure 8C, the MIC value was significantly decreased for both PDA-CUR-MOFs and CUR-Zn-MOFs, which were determined to be 9.09 ± 0.6 and 13.20 ± 0.6 µg/mL, respectively. CUR had an IC50 of 76.92 ± 0.5 µg/mL against E. coli cells, while Zn-MOFs had an IC50 of 100 ± 0.5 µg/mL; however, the value for cells treated with CUR-Zn-MOFs was reduced to 66.03 ± 0.6% µg/mL, and for PDA-coated CUR-Zn-MOFs was further decreased to 45.45 ± 0.6 µg/mL. When PDA was coated onto CUR-Zn-MOFs, the IC50 value of all of the tested bacterial strains was further reduced as shown in Figure 8D. The previously reported MIC value for CUR against S. aureus and E. coli was determined to be 100 µg/mL (Gao et al., 2020). The antibacterial activity of PDA, which was measured to be 90 µg/mL against both S. aureus and E. coli was not promising in a prior study published by Indu Singh., et al. However, the PDA surface modification of the nanocomposites significantly reduced the growth of bacteria with a MIC value of less than 30 µg/mL against both strains (Zhang et al., 2022). The increased antibacterial activity of PDA-CUR-Zn-MOFs was attributed to the fact that PDA may facilitate drug permeation into bacterial cells by enhancing cell permeability. Furthermore, ROS (reactive oxygen species), including the well-known disinfectants hydrogen peroxide (H2°2) and superoxide anions (O2-), formed by the oxidation of catechol moieties hindered the growth of bacteria (Zhang et al., 2022). Thus, the study found that the bactericidal activity of CUR was much enhanced when it was encapsulated in PDA-coated Zn-MOFs. This is because the interaction between PDA coatings and surface characteristics can considerably alter the antibacterial properties (Singh et al., 2021).

#### 3.6.2. Assessment of MBIC value

**Figure 9.**
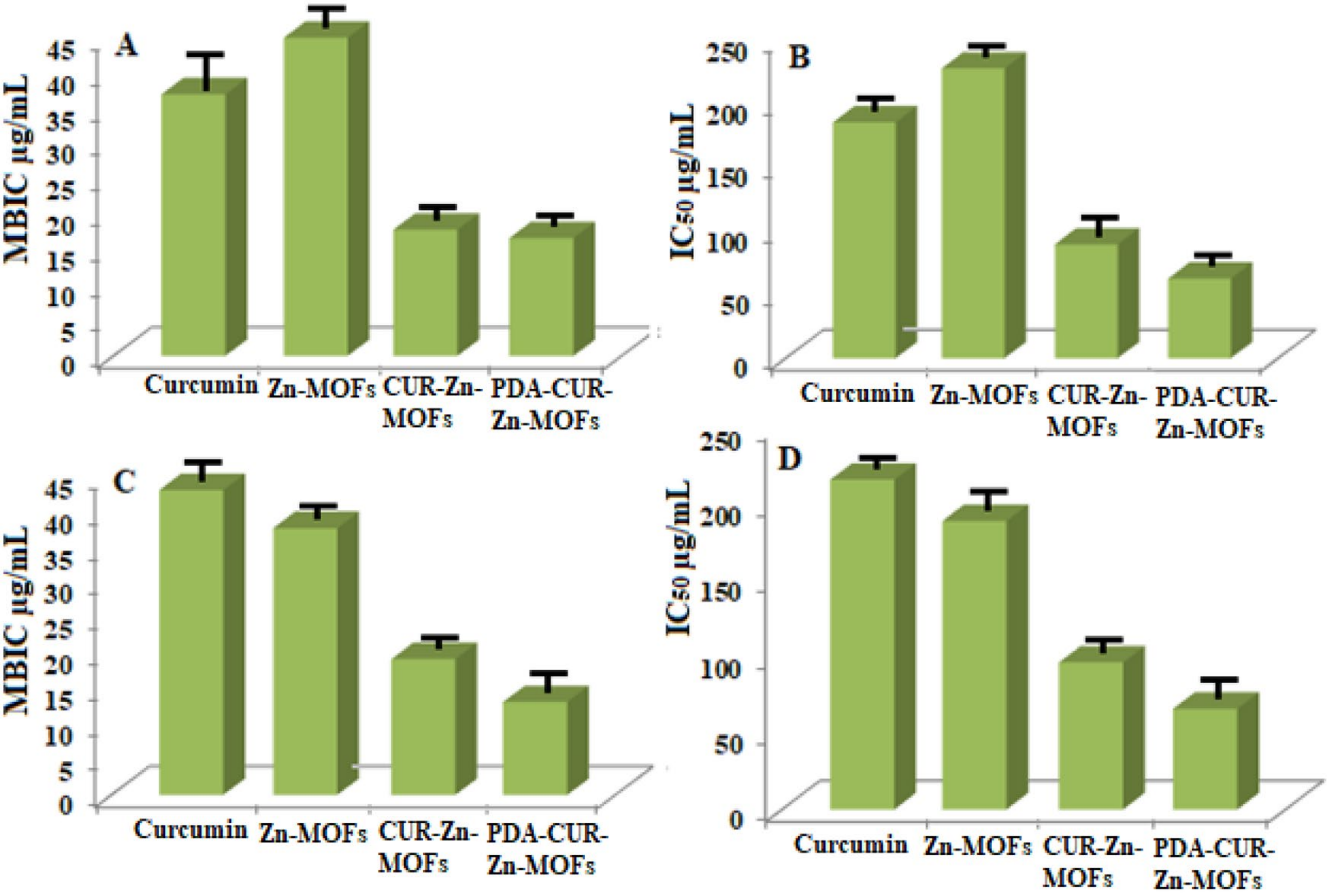
MBIC and IC50 value of CUR, Zn-MOFs, CUR-Zn-MOFs and PDA-CUR-Zn-MOFs against S. aureus (A, B), and against E. coli (C, D).

Biofilms are the most common kind of microbial life because they allow bacteria to survive in harsh environments (Taylor et al., 2014). Antibiotic-resistant bacteria often thrive in biofilm environments (Dantas et al., 2008). Consequently, the MBIC value of prepared Zn-MOFs was also studied against both strains. For S. aureus, the MBIC for CUR was 37 ± 0.7 µg/mL, while the MBIC for Zn-MOFs was 45 ± 1.2 µg/mL. CUR’s MBIC was decreased to 17.85 ± 0.5 µg/mL and 16.66 ± 0.6 µg/mL after being encapsulated in Zn-MOFs and modified with PDA, respectively (Figure 9A). CUR had an IC_50_ of 185 ± 0.7 µg/mL against S. aureus cells, while Zn-MOFs had a greater IC_50_ of 227.27 ± 1.2 µg/mL against S. aureus, while CUR-Zn-MOFs had an IC_50_ of 89.28 ± 0.5 µg/mL, which was further decreased to 62.5 ± 0.6 µg/mL after PDA coating, as displayed in Figure 9B.

The MIC values for CUR and Zn-MOFs against E. coli were 43.10 ± 0.8 µg/mL and 37.73 ± 0.5 µg/mL, respectively (Figure 9C). After CUR loading and PDA coating on the prepared Zn-MOFs, these values were dropped to 19.23 ± 0.4 µg/mL and 13.15 ± 0.6 µg/mL, respectively. The IC_50_ values for CUR against E. coli were 215.5 ± 0.8 µg/mL, 188.5 ± 0.5 µg/mL for Zn-MOFs, 996.1 ± 0.4 µg/mL for CUR-Zn-MOFs, and 65.78 ± 0.6 µg/mL for PDA coated CUR-Zn-MOFs (Figure 9D). Increased antibacterial property of CUR in PDA-CUR-Zn-MOFs was caused by the interaction of PDA coating, which are capable to regulate surface properties and may have a major impact on antibacterial activities (Singh et al., 2021).

#### 3.6.3. Surface morphological study

**Figure 10.**
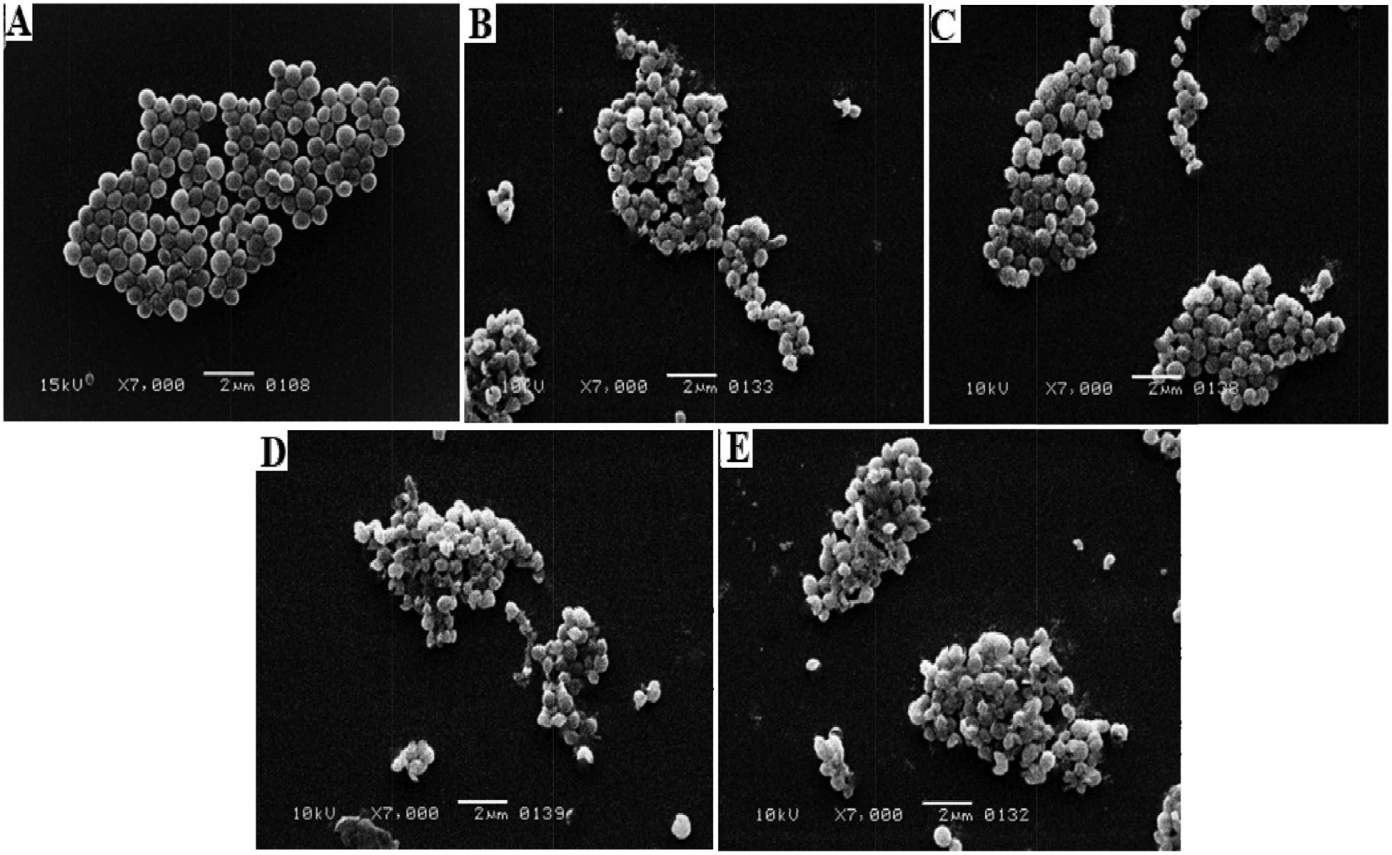
S. aureus control (A), S. aureus CUR treated (B), S. aureus Zn-MOFs treated (C), S. aureus CUR-Zn-MOFs treated (D), and S. aureus PDA- CUR-Zn-MOFs treated (E).

**Figure 11.**
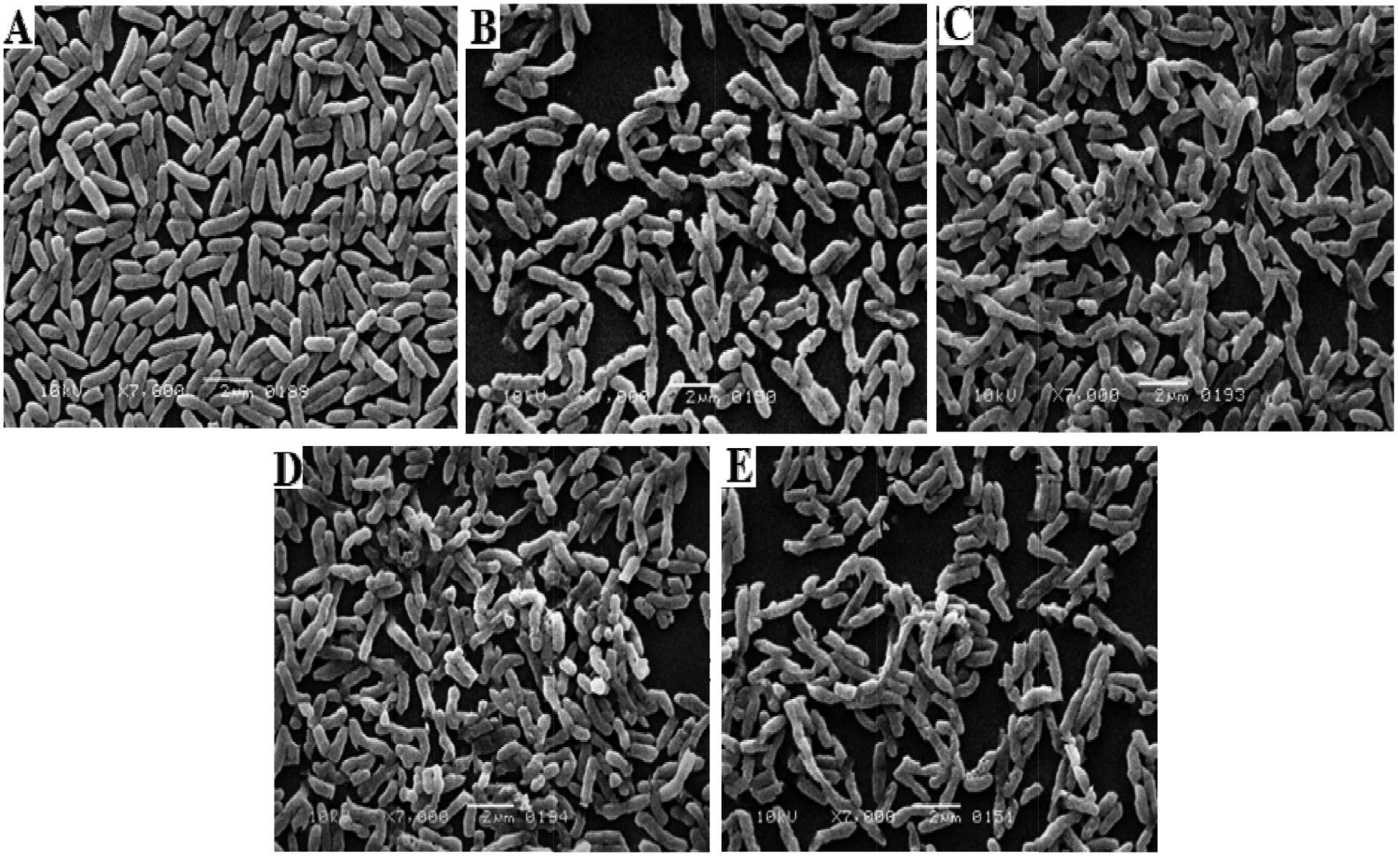
E. coli control (A), E. coli CUR treated (B), E. coli Zn-MOFs treated (C), E. coli CUR-Zn-MOFs treated (D), and E. coli PDA- CUR-Zn-MOFs treated (E).

Morphological studies of both E. coli and S. aureus strains were performed using SEM to evaluate the effectiveness of the synthesized MOFs. The morphological alterations that occurred in the bacterial strains after being exposed to the prepared samples are shown in Figures 10 and 11. S. aureus control group morphology was identified as a smooth spherical shape with an agglomerated colony as displayed in Figure 10A. Following treatment with CUR, S. aureus cells lost some of their surface smoothness, and the colony became disturbed, but after treatment with Zn-MOFs, the cells kept their morphology while the agglomerated colony somewhat distasted as displayed in Figure 10B and C. Minor changes in bacterial morphology were observed after treatment with CUR-Zn-MOFs (Figure 10D), but significant changes were observed when the cells were exposed to PDA-CUR-Zn-MOFs (Figure 10E). Synergistic improvement of CUR’s effectiveness against S. aureus may be attributed to PDA’s tendency to modify membrane permeability and bacterial cell destruction (Iqbal et al., 2012).

Similarly, treatment of the E. coli strain with all of the test samples exhibited similar results, with the bacterial strains showing morphological alterations. Figure 11A depicts the smooth rod-like surface morphology of the control E. coli strain, whereas Figure 11B and C illustrate the effects of CUR and Zn-MOFs on the morphological characteristics of the bacterial strains, respectively. Figure 11D and 11E show that after being exposed to Cur-Zn-MOFs and PDA-CUR-Zn-MOFs, the rod-shaped bacterial cells were completely distorted (Khan et al., 2021). This may be attributed to the generation of ROS by the catechol moieties of PDA, which may have triggered the obstruction of internal matrix in the external environment by attaching to the bacterial cell surface, leading to cell disruption (Guo et al., 2014). According to a recent study, PDA prevents bacteria from extruding metabolic waste and absorbing nutrients, which has an immediate impact on the survival of the bacteria (Iqbal et al., 2012). These findings demonstrated conclusively that PDA’s excellent bio-adhesive activity significantly contributed to an efficient bacterial cell growth distortion.

## 4. Conclusion

In this work, we have successfully prepared Zn-MOFs and modified them with a biocompatible polymer (PDA). The prepared MOFs were capable of encapsulating excess amount of CUR, and exhibited enhanced antibacterial activity of CUR against E. coli and S. aureus. Interestingly, surface modification with PDA notably decreased the MIC and MBIC value of CUR-Zn-MOFs. The findings were further supported by analyzing the surface morphology via SEM, which exhibited the complete distortion of bacterial cells after treatment with PDA-CUR-Zn-MOFs. Conclusively, this study demonstrated that the PDA-coated Zn-MOFs have the potential to control the MDR of bacteria, making them a potential antibacterial alternative for use in preclinical settings for the cure of wide range of bacterial infections.

## Data Availability

The data supporting this work are accessible upon reasonable request from the corresponding author.
